# Clinical biomarker-based biological ageing and future risk of neurological disorders in the UK Biobank

**DOI:** 10.1136/jnnp-2023-331917

**Published:** 2023-11-05

**Authors:** Jonathan K L Mak, Christopher E McMurran, Sara Hägg

**Affiliations:** 1 Department of Medical Epidemiology and Biostatistics, Karolinska Institutet, Stockholm, Sweden; 2 Department of Clinical Neurosciences, University of Cambridge, Cambridge, UK

**Keywords:** STROKE, MOTOR NEURON DISEASE, PARKINSON'S DISEASE, VASCULAR DEMENTIA, ALZHEIMER'S DISEASE

## Abstract

**Background:**

Many common neurological disorders are associated with advancing chronological age, but their association with biological age (BA) remains poorly understood.

**Methods:**

We studied 325 870 participants in the UK Biobank without a diagnosed neurological condition at baseline and generated three previously-described measures of BA based on 18 routinely measured clinical biomarkers (PhenoAge, Klemera-Doubal method age (KDMAge), homeostatic dysregulation age). Using survival models, we assessed the effect of advanced BA on incident neurological diagnoses, including all-cause and cause-specific dementia, ischaemic stroke, Parkinson’s disease and motor neuron disease.

**Results:**

During a mean follow-up of 9.0 years, there were 1397 incident cases of dementia and 2515 of ischaemic stroke, with smaller case numbers of other diagnoses. The strongest associations with a 1 SD in BA residual were seen for all-cause dementia (KDMAge HR=1.19, 95% CI=1.11 to 1.26), vascular dementia (1.41, 1.25 to 1.60) and ischaemic stroke (1.39, 1.34 to 1.46). Weaker associations were seen for Alzheimer’s disease and motor neuron disease, while, in contrast, HRs for Parkinson’s disease tended to be <1. Results were largely consistent after adjustment for disease-specific covariates including common cardiometabolic risk factors.

**Conclusions:**

Advanced BA calculated from routine clinical biomarker results increases the risk of subsequent neurological diagnoses including all-cause dementia and ischaemic stroke.

## Introduction

Advancing age is a principal risk factor for many of the most common neurological disorders. Measures of biological age (BA), such as telomere length, epigenetic clocks and composite biomarker predictors, have been developed to represent the heterogeneity in how people age and explain age-associated outcomes in a more nuanced way than chronological age (CA; time since birth).^1^ Some of the most clinically relevant approaches to measuring BA harness variation in routinely measured clinical biomarkers, with previous work demonstrating that when such BA measures are higher than would be expected for one’s CA, this increases the risk of mortality,^2^ several cancers^3^ and depression/anxiety.^4^ However, few studies have assessed the associations between these BA measures and the risk of neurological disorders, and they often have limited power to examine the less common diagnoses.^5 6^

Building on these findings, the current study examines the association between three previously described clinical biomarker-based measures of BA and the subsequent risk of age-related neurological diagnoses among over 300 000 participants in the UK Biobank. We report and compare the effect of advanced BA on time-to-event models for all-cause and cause-specific dementia, ischaemic stroke, Parkinson’s disease (PD) and motor neuron disease (MND).

## Methods

### Participants

We carried out a prospective cohort analysis in the population-based UK Biobank, which recruited >500 000 volunteers aged 37–73 years between 2006 and 2010.^7^ During baseline assessment, participants completed a questionnaire, had physical and functional measurements taken and provided biological samples. After excluding those who had missing data on the BA measures and the common covariates, or had a pre-existing dementia, ischaemic stroke, PD or MND, we included 325 870 participants in the analyses (online supplemental figure 1).

10.1136/jnnp-2023-331917.supp1 Supplementary data

### Biological age

Three clinical biomarker-based BA measures were previously derived in the UK Biobank, with the full methods described elsewhere.^3^ Briefly, we selected 18 age-related clinical biomarkers that correlate with CA for construction of the BA algorithms (online supplemental figure 2). Using data from the US National Health and Nutrition Examination Surveys, we combined information from the biomarkers and trained and validated three composite measures of BA, namely Klemera-Doubal method age (KDMAge),^8^ PhenoAge^9^ and homeostatic dysregulation age (HDAge).^10^ KDMAge is computed based on regression models of biomarkers on age and represents the predicted physiological age of an individual. PhenoAge is trained based on a mortality prediction score of biomarkers which captures information not only on CA, but also mortality risk. We regressed KDMAge and PhenoAge on CA (as a 3 df natural spline), such that the resulting residual values can be interpreted as the deviation between BA and CA. By contrast, HDAge is not an age measure by definition, but it is calculated as the deviation of an individual’s physiology from a healthy reference sample. HDAge was log-transformed before analysis due to its skewed distribution.

### Outcomes

Incident cases of neurological disorders were ascertained based on the International Classification of Diseases, 10th revision codes obtained from hospital inpatient and death register records, including all-cause dementia (A81.0, F00–F03, F05.1, F10.6, G30, G31.0, G31.1, G31.8), Alzheimer’s disease (F00, G30), vascular dementia (F01, I67.3), ischaemic stroke (I63), PD (G20) and MND (G12.2). Participants with a diagnosis or a self-reported history of neurological disorders before baseline were excluded from the analysis.

### Statistical analysis

We used Cox proportional-hazards models, where the follow-up time was calculated from date of baseline assessment to date of disease diagnosis, death or end of follow-up (1 April 2018), whichever came first. Attained age was used as the underlying timescale. Models were first adjusted for birth year and sex, and were further adjusted for baseline assessment centre, ethnicity, body mass index, smoking, alcohol and deprivation as the common covariates for all outcomes (multivariable model). We also included models with disease-specific covariates, which were selected based on the literature and the data availability (online supplemental table 1).^11–14^ We tested the proportional hazard assumption of the models using Schoenfeld residuals and found no violations. The false discovery rate method was used to correct for multiple testing (corrected significance level at 0.034). All analyses were conducted using R V.4.2.3.

## Results

The study sample included 325 870 participants (mean age 56.4; 54.2% women). During a mean follow-up of 9.0 years, 1397 (0.4%), 2515 (0.8%), 679 (0.2%) and 203 (0.1%) participants were diagnosed with dementia, ischaemic stroke, PD and MND, respectively (table 1).

**Table 1:** Baseline characteristics of participants (n=325 870)

Characteristic	N (%) or mean±SD
Age (years)	56.4±8.1
Sex	
Women	176 631 (54.2)
Men	149 239 (45.8)
Baseline assessment centre	
England	299 083 (91.8)
Wales	14 337 (4.4)
Scotland	12 450 (3.8)
Ethnicity	
White	309 556 (95.0)
Asian	7218 (2.2)
Black	4384 (1.3)
Others	4712 (1.4)
Body mass index	
<18.5	1619 (0.5)
18.5 to <25	107 682 (33.0)
25 to <30	139 343 (42.8)
≥30	77 226 (23.7)
Smoking status	
Never	179 962 (55.2)
Previous	112 699 (34.6)
Current	33 209 (10.2)
Biological age measures	
KDMAge (years)	54.12±9.42
KDMAge residual	-0.03±5.01
PhenoAge (years)	47.63±10.02
PhenoAge residual	-0.03±5.37
HDAge (log units)	6.70±1.00
Died during follow-up	12 144 (3.7)
Incident all-cause dementia during follow-up	1397 (0.4)
Incident Alzheimer’s disease during follow-up	557 (0.2)
Incident vascular dementia during follow-up	297 (0.1)
Incident ischaemic stroke during follow-up	2515 (0.8)
Incident Parkinson’s disease during follow-up	679 (0.2)
Incident motor neuron disease during follow-up	203 (0.1)

HDAge, homeostatic dysregulation age; *KDMAge*, Klemera-Doubal method age.

**Figure 1:**
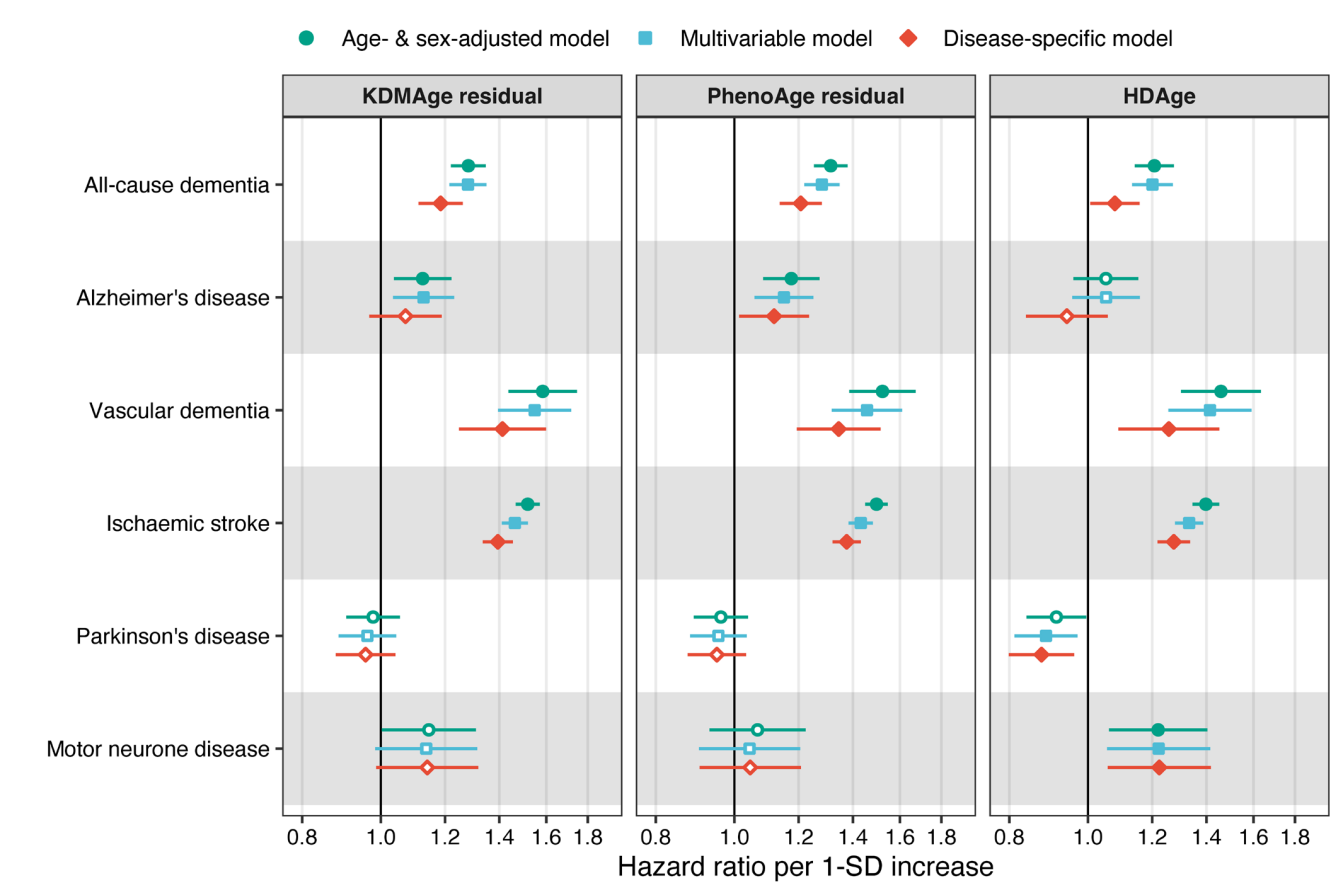
HRs and 95% CIs for neurological disorders in relation to 1 SD increase in biological age measures. Filled symbols represent statistically significant associations at a false discovery rate corrected significance level of 0.034. Age-adjusted and sex-adjusted models were adjusted for age (timescale), birth year and sex, and multivariable models were additionally adjusted for baseline assessment centre, ethnicity, body mass index, smoking, alcohol consumption and deprivation (n=325 870). Disease-specific models further included covariates that are relevant for each outcome based on the literature. The models for all-cause dementia, Alzheimer’s disease and vascular dementia included education, physical activity, social isolation, air pollution, diabetes, hypertension, depressive symptoms, hearing impairment, traumatic brain injury, *APOE* e4 allele and family history of dementia (n=269 290). The models for ischaemic stroke included physical activity, air pollution, fresh vegetable and fruit intake, red meat intake, processed meat intake, diabetes, hypertension, depressive symptoms, dyslipidaemia, atrial fibrillation and family history of stroke (n=280 433). The models for Parkinson’s disease included hypertension, depressive symptoms, traumatic brain injury and family history of Parkinson’s disease (n=310 866). The models for motor neuron disease included family history of dementia (n=325 870). Details of the covariate definitions are shown in online supplemental table 1. APOE, apolipoprotein E; HDAge, homeostatic dysregulation age; KDMAge, Klemera-Doubal method age.

Estimates of the association between a 1 SD increase in BA and neurological disorders are shown in figure 1 and online supplemental table 2. After adjusting for the common covariates, KDMAge residual (HR=1.28, 95% CI=1.21 to 1.35), PhenoAge residual (1.28, 1.22 to 1.35) and HDAge (1.20, 1.13 to 1.27) were all statistically significantly associated with an increased future risk of all-cause dementia. The estimates remained significant when further adjusting for dementia-specific covariates. When stratified by dementia subtypes, all BA measures were strongly associated with vascular dementia, while weaker associations were seen for Alzheimer’s disease. Similarly, there was a statistically significantly increased risk of BA measures on ischaemic stroke: KDMAge residual (HR=1.39, 95% CI=1.34 to 1.46), PhenoAge residual (1.38, 1.32 to 1.43) and HDAge (1.28, 1.22 to 1.34). There were weak positive associations between advanced BA and subsequent risk of MND, but only HDAge was statistically significantly associated with MND (HR 1.22, 95% CI 1.06 to 1.42). There were similarly no significant associations between BA residuals and PD risk, however, unlike all other outcomes studied, the HRs for PD tended to be below 1.0: KDMAge residual (HR=0.96, 95% CI=0.88 to 1.04), PhenoAge residual (0.95, 0.88 to 1.03) and HDAge (0.88, 0.80 to 0.93).

Results were largely similar when stratified by age and sex, although the association between BA and dementia appeared to be stronger in younger participants aged <60 years and in women (online supplemental table 3). Several of the individual biomarkers included in the BA measures were predictive for specific neurological outcomes. For example, a higher forced expiratory volume was associated with lower risks of dementia and ischaemic stroke, whereas a higher red blood cell count was associated with increased risks of dementia and PD (online supplemental figure 2). Meanwhile, BA measures appeared to have a more global predictive effect across different neurological outcomes.

Finally, to mitigate against BA assessments taking place after disease onset but prior to formal diagnosis, we repeated the analysis excluding individuals that were diagnosed within 5 years following their BA assessment (online supplemental table 4). Effect sizes were generally smaller but remained in the same direction, with significant associations remaining for all-cause and vascular dementia, ischaemic stroke and for PhenoAge in Alzheimer’s disease.

## Discussion

In this population-based study, we found that advanced biological ageing is associated with increased risk for several age-associated neurological diagnoses, with the largest effect sizes seen for all-cause dementia, vascular dementia and ischaemic stroke. Some of this increased risk will be a consequence of the selection of biomarkers used in the BA measures, many of which reflect cardiometabolic health and are independently associated with stroke and all-cause dementia (online supplemental figure 2). That said, strong associations remained after adjusting for relevant confounders in the disease-specific models (including the presence of hypertension, diabetes, dyslipidaemia and smoking history), suggesting that these BA measures have utility beyond simply cardiovascular risk prediction. Our findings are largely consistent with a previous analysis of the Rotterdam Study (n=1930, mean age 72), which used variations of phenotypic age built on a different but overlapping panel of BA biomarkers.^5^ The Rotterdam Study reported a similar effect size of BA residual on ischaemic stroke risk, although effect on all-cause dementia risk was only seen with the addition of a central nervous system-specific biomarker (neurofilament light chain), highlighting the importance of comparing BA measures across different cohorts.^6^

The unparalleled scale of the UK Biobank cohort also allows for interrogation of less common age-associated neurological outcomes. Advanced BA measures have a small positive effect size on MND risk, although with wide CIs. These estimates are of a similar size to those seen for Alzheimer’s disease, another age-associated neurodegenerative disease characterised by protein aggregation. Interestingly, similar to the results found in a previous study,^6^ the effect sizes in PD were in the opposite direction to our other outcomes: advanced BA does not appear to increase PD risk and if anything may be protective. Estimates were similar despite adjustment for smoking history, which might drive higher BA while simultaneously reducing PD risk.^13^ Alternatively, the apparent protective effect could be driven by the individual biomarkers included in the BA measures, such as systolic blood pressure and uric acid that were negatively associated with PD risk but positively associated other neurological outcomes online supplemental figure 2. While blood pressure and serum uric acid levels usually increased with advancing age,^15 16^ orthostatic hypotension and reduced serum uric acid levels have been associated with a higher risk of PD diagnosis.^17 18^

Comparison between the different BA measures is informative, given there is currently no gold standard approach to calculating BA from clinical biomarkers. For example, Alzheimer’s disease—a common cause of death at the population level—had a relatively strong association with PhenoAge, a BA measure based on mortality risk. In contrast, PD and MND were more associated with HDAge, which is based on biomarker deviation from a young reference population. HDAge is naïve to the direction of biomarker deviation,^10^ some of which would be expected to move in the opposite direction to ‘normal’ ageing in specific pathologies, for example, people with MND tend to have low serum creatinine related to muscle atrophy.^19^ Some of these associations might therefore have contributions from more disease-specific biomarker signatures during a prodromal period prior to diagnosis.

Strengths of this study include the large sample size, which provides statistical power to examine some less common neurological disorders while adjusting for multiple confounding factors. Nevertheless, it should be acknowledged that UK Biobank is a relatively healthy cohort in comparison to the general UK population due to a ‘healthy volunteer’ selection bias.^20^ While we know something about the temporal relationship between BA advancement and diagnosis (participants did not have a neurological diagnosis when their BA was assessed, and diagnoses were collected over a mean follow-up of 9.0 years), as an observational study we cannot establish causal relationships. As alluded to, the insidious onset of neurodegenerative disorders is a particular consideration, with some of the BA assessments likely occurring during a prodromal period and our ascertainment of neurological conditions relied solely on medical records. We have mitigated against this with sensitivity analysis in which participants were excluded if their diagnosis was made within 5 years following their BA assessment. Further follow-up of the UK Biobank cohort in the coming years will further help to clarify some of these points.

In summary, in a large population study, we find that higher measures of BA derived from routine clinical biomarkers increase one’s risk for dementia or stroke, despite adjustment for disease-specific risk factors.
