# Supplementary material for: Clinical biomarker-based biological ageing and future risk of neurological disorders in the UK Biobank
Source: J Neurol Neurosurg Psychiatry. 2023 Nov 5;95(5):481–4. doi: 10.1136/jnnp-2023-331917 (PMC11041565; doi:10.1136/jnnp-2023-331917)
Supplement: Supplementary data [file jnnp-2023-331917supp001.pdf]

Supplementary Figure 1. Flowchart for the selection of participants.

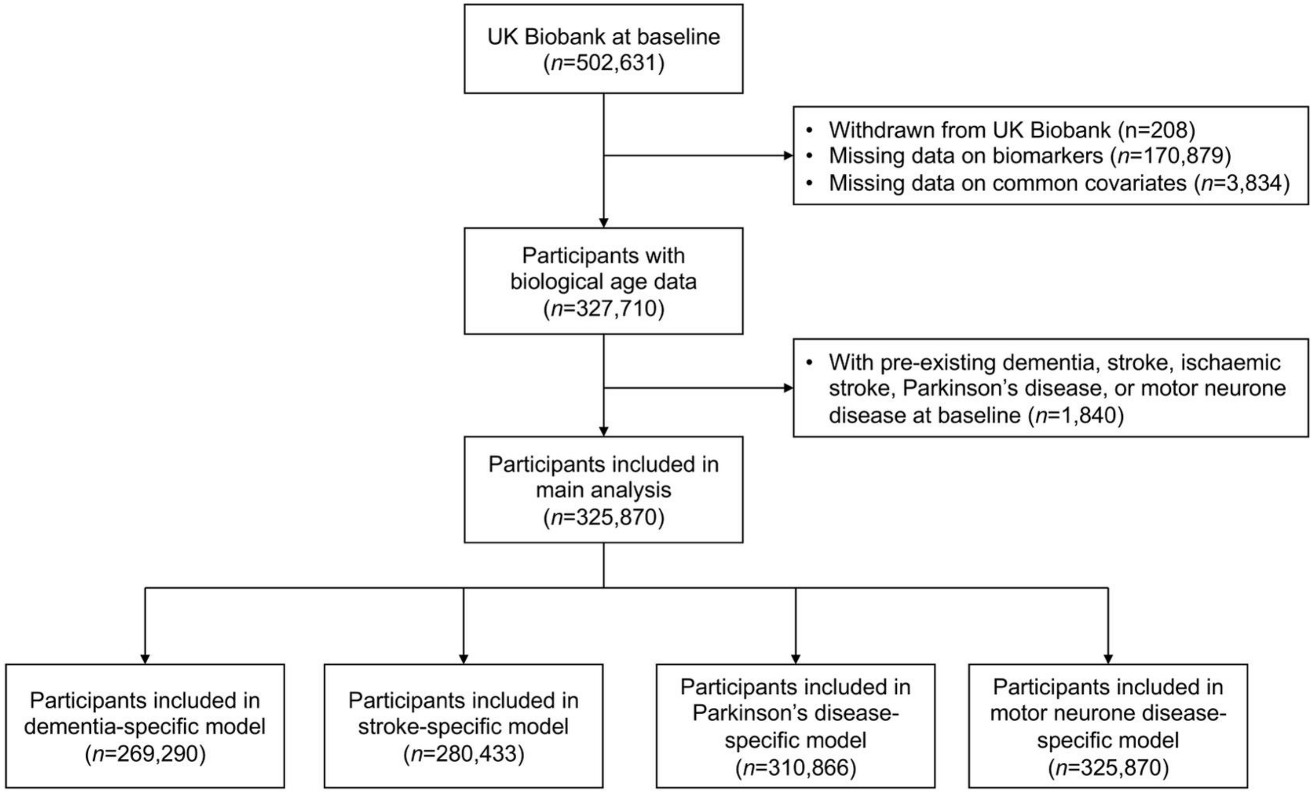

Supplementary Figure 2. Hazard ratios and 95% confidence intervals for neurological disorders in relation to 1-SD increase in biological age measures and the 18 individual clinical biomarkers.

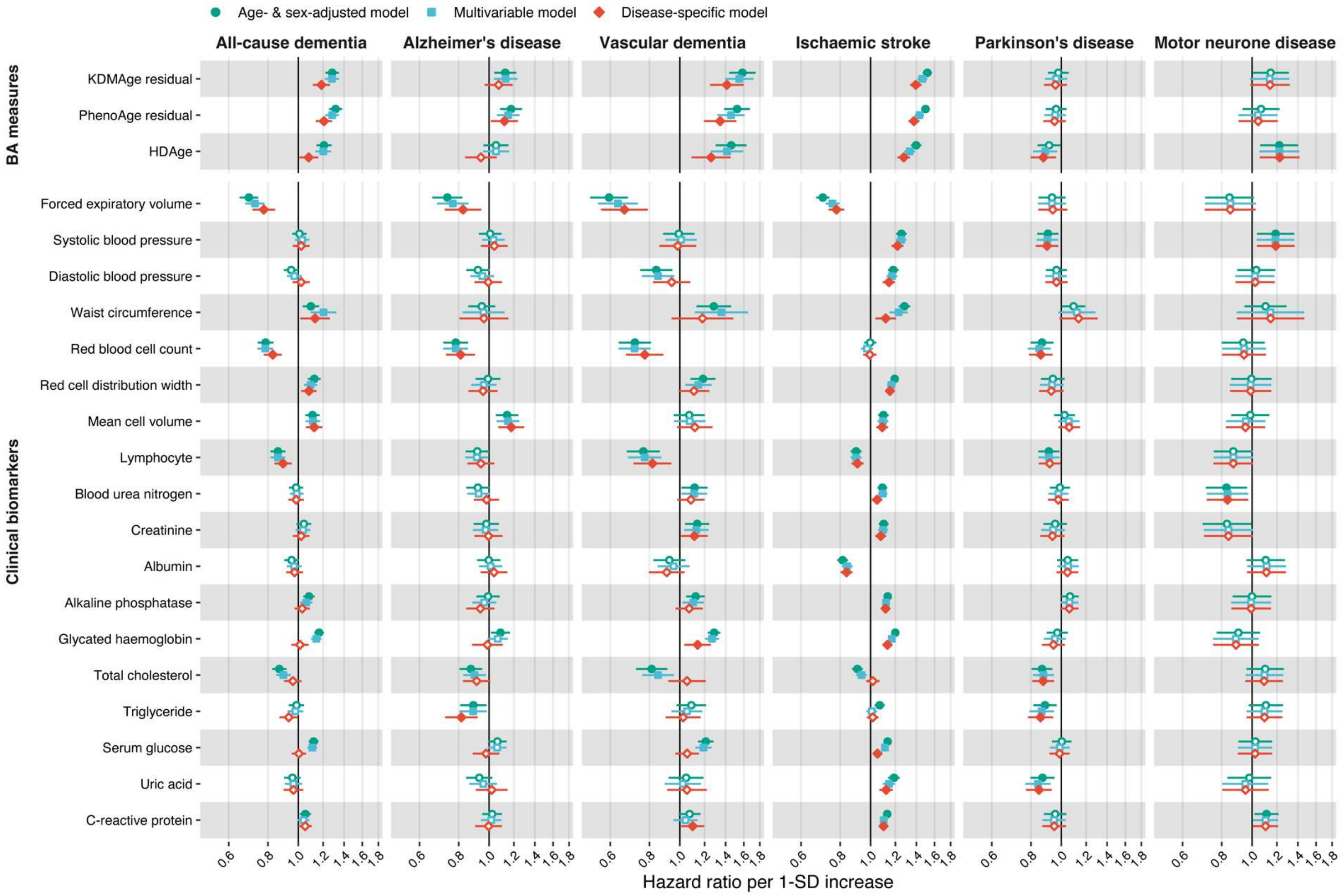

Filled symbols represent statistically significant associations a false discovery rate corrected significance level of 0.034. *HDAge*, homeostatic dysregulation age; *KDMAge*, Klemera-Doubal method age; *SD*, standard deviation.

Supplementary Table 1. Definitions of the covariates included in the disease-specific models

Covariate	Description	Categorization	Field ID	Dementia-specific model	Stroke-specific model	Parkinson's disease-specific model	Motor neurone disease-specific model
**Age (timescale)**	Date of baseline assessment minus date of birth	Continuous	53, 34, 52	Yes	Yes	Yes	Yes
**Year of birth**	Year of birth	1930–1939; 1940–1949; 1950–1959; ≥1960	34	Yes	Yes	Yes	Yes
**Sex**	Self-reported sex or NHS-derived	Women; Men	31	Yes	Yes	Yes	Yes
**Assessment centre**	UK Biobank assessment centre	England; Wales; Scotland	54	Yes	Yes	Yes	Yes
**Ethnicity**	Self-reported ethnic background	White; Asian; Black; Others	21000	Yes	Yes	Yes	Yes
**Body mass index**	Body mass index calculated from measured height and weight	Underweight (<18.5); Normal weight (18.5 to <25); Overweight (25 to <30); Obese (≥30)	21001	Yes	Yes	Yes	Yes
**Smoking**	Self-reported smoking status	Never; Previous; Current	20116	Yes	Yes	Yes	Yes
**Alcohol**	Self-reported alcohol intake frequency	Less than 3 times a month; 1–4 times a week; Daily or almost daily	1558	Yes	Yes	Yes	Yes
**Deprivation**	Townsend deprivation index derived from national census data	Quintiles of deprivation index	22189	Yes	Yes	Yes	Yes
**Education**	Self-reported highest qualifications	Low (no relevant qualifications); Intermediate (A levels, O levels/GCSEs, CSEs, NVQ/HND/HNC, other professional qualifications); High (college or university degree)	6138	Yes	-	-	-
**Physical activity**	Physical Activity Questionnaire activity group	Low; Moderate; High	22032	Yes	Yes	-	-
**Social isolation**	Defined based on three questions: (1) "Including yourself, how many people are living together in your household?" (1 point if living alone); (2) "How often do you visit friends or family or have them visit you?" (1 point if less than once a month); (3) "Which of the following [leisure/social activities] do you engage in once a week or more often?" (1 point if no activity)	No (total score <2); Yes (total score ≥2)	709, 1031, 6160	Yes	-	-	-
**Air pollution**	Particulate matter air pollution ($PM_{2.5}$)	Continuous ($\mu g/m^3$)	24006	Yes	Yes	-	-
**Fresh vegetable and fruit intake**	Self-reported intake frequency of salad/raw vegetables and fresh fruits	<5 portions a day; ≥5 portions a day	1299, 1309	-	Yes	-	-
**Red meat intake**	Self-reported intake frequency of beef, lamb, and pork	Less than twice a week; Twice a week or more	1369, 1379, 1389	-	Yes	-	-
**Processed meat intake**	Self-reported intake frequency of processed meat	Less than twice a week; Twice a week or more	1349	-	Yes	-	-
**Diabetes**	Self-reported diabetes	No; Yes	2443, 20002	Yes	Yes		-
**Hypertension**	Self-reported high blood pressure	No; Yes	6150, 20002	Yes	Yes	Yes	-
**Depressive symptoms**	Self-reported frequency of depressed mood in last 2 weeks	Not at all; Several days; More than half the days; Nearly every day	2050	Yes	Yes	Yes	-

Supplementary Table 1. (*continued*)

Covariate	Description	Categorization	Field ID	Dementia-specific model	Stroke-specific model	Parkinson's disease-specific model	Motor neurone disease-specific model
**Hearing impairment**	Self-reported hearing difficulty/problems	No; Yes	2247	Yes	-		-
**Traumatic brain injury**	Self-reported head injury	No; Yes	20002	Yes	-	Yes	-
**Dyslipidaemia**	Self-reported use of cholesterol lowering medication	No; Yes	6153, 6177	-	Yes	-	-
**Atrial fibrillation**	Self-reported atrial fibrillation	No; Yes	20002	-	Yes	-	-
***ApoE* e4 allele**	Determined based on two single nucleotide polymorphisms: rs7412 and rs429358	None (e2e2, e2e3 or e3e3), One (e3e4 or e2e4), and Two (e4e4)	rs7412, rs429358	Yes	-	-	-
**Family history of dementia**	Self-reported illnesses in father, mother, or siblings	No; Yes	20107, 20110, 20111	Yes	-	-	Yes
**Family history of Parkinson's disease**	Self-reported illnesses in father, mother, or siblings	No; Yes	20107, 20110, 20111	-	-	Yes	-
**Family history of stroke**	Self-reported illnesses in father, mother, or siblings	No; Yes	20107, 20110, 20111	-	Yes	-	-

Supplementary Table 2. Association between biological age measures and neurological disorders

Model	KDMAge residual		PhenoAge residual		HDAge	
	HR per SD increase (95% CI)	*p*	HR per SD increase (95% CI)	*p*	HR per SD increase (95% CI)	*p*
**All-cause dementia**						
Age- & sex-adjusted model[a]	1.28 (1.22, 1.35)*	1.0×10^{-22}	1.31 (1.25, 1.38)*	4.8×10^{-29}	1.21 (1.14, 1.28)*	3.1×10^{-11}
Multivariable model[b]	1.28 (1.21, 1.35)*	4.1×10^{-20}	1.28 (1.22, 1.35)*	3.0×10^{-22}	1.20 (1.13, 1.27)*	6.0×10^{-10}
Disease-specific model[c]	1.19 (1.11, 1.26)*	1.1×10^{-7}	1.21 (1.14, 1.28)*	6.9×10^{-10}	1.08 (1.01, 1.16)*	0.033
**Alzheimer's disease**						
Age- & sex-adjusted model[a]	1.13 (1.04, 1.22)*	0.004	1.18 (1.08, 1.27)*	7.8×10^{-5}	1.05 (0.96, 1.15)	0.281
Multivariable model[b]	1.13 (1.04, 1.23)*	0.006	1.15 (1.06, 1.25)*	0.001	1.05 (0.96, 1.16)	0.296
Disease-specific model[c]	1.07 (0.97, 1.19)	0.181	1.12 (1.01, 1.24)*	0.026	0.94 (0.84, 1.06)	0.312
**Vascular dementia**						
Age- & sex-adjusted model[a]	1.58 (1.44, 1.75)*	2.0×10^{-20}	1.52 (1.39, 1.67)*	2.4×10^{-18}	1.46 (1.30, 1.63)*	7.6×10^{-11}
Multivariable model[b]	1.55 (1.39, 1.72)*	1.8×10^{-16}	1.46 (1.32, 1.61)*	1.6×10^{-13}	1.41 (1.26, 1.59)*	9.0×10^{-9}
Disease-specific model[c]	1.41 (1.25, 1.60)*	4.6×10^{-8}	1.34 (1.19, 1.51)*	1.1×10^{-6}	1.26 (1.09, 1.45)*	0.002
**Ischemic stroke**						
Age- & sex-adjusted model[a]	1.52 (1.47, 1.57)*	5.9×10^{-127}	1.50 (1.45, 1.55)*	1.5×10^{-133}	1.40 (1.35, 1.45)*	3.6×10^{-66}
Multivariable model[b]	1.46 (1.41, 1.52)*	1.3×10^{-89}	1.43 (1.38, 1.48)*	2.1×10^{-91}	1.33 (1.28, 1.39)*	2.8×10^{-44}
Disease-specific model[c]	1.39 (1.34, 1.46)*	3.4×10^{-52}	1.38 (1.32, 1.43)*	6.2×10^{-55}	1.28 (1.22, 1.34)*	4.4×10^{-25}
**Parkinson's disease**						
Age- & sex-adjusted model[a]	0.98 (0.91, 1.06)	0.575	0.96 (0.89, 1.04)	0.329	0.91 (0.84, 1.00)	0.038
Multivariable model[b]	0.96 (0.89, 1.04)	0.362	0.96 (0.88, 1.04)	0.270	0.89 (0.81, 0.97)*	0.009
Disease-specific model[c]	0.96 (0.88, 1.04)	0.318	0.95 (0.88, 1.03)	0.242	0.88 (0.80, 0.96)*	0.005
**Motor neurone disease**						
Age- & sex-adjusted model[a]	1.15 (1.00, 1.31)	0.046	1.07 (0.93, 1.22)	0.344	1.22 (1.06, 1.40)*	0.005
Multivariable model[b]	1.14 (0.98, 1.32)	0.081	1.04 (0.90, 1.21)	0.557	1.22 (1.06, 1.42)*	0.007
Disease-specific model[c]	1.14 (0.99, 1.32)	0.074	1.05 (0.91, 1.21)	0.541	1.22 (1.06, 1.42)*	0.007

CI, confidence interval; *HDAge*, homeostatic dysregulation age; *HR*, hazard ratio; *KDMAge*, Klemera-Doubal method age; *SD*, standard deviation.

[a] Age- & sex-adjusted models: adjusted for age (timescale), birthyear, and sex (n=325,870).

[b] Multivariable models: additionally adjusted for baseline assessment centre, ethnicity, body mass index, smoking, alcohol consumption, and deprivation (n=325,870).

[c] Disease-specific models further included covariates that are relevant for each outcome based on the literature (**Supplementary Table 1**). The models for all-cause dementia, Alzheimer's disease, and vascular dementia included education, physical activity, social isolation, air pollution, diabetes, hypertension, depressive symptoms, hearing impairment, traumatic brain injury, *ApoE* e4 allele, and family history of dementia (n=269,290). The models for ischaemic stroke included physical activity, air pollution, fresh vegetable and fruit intake, red meat intake, processed meat intake, diabetes, hypertension, depressive symptoms, dyslipidaemia, atrial fibrillation, and family history of stroke (n=280,433). The models for Parkinson's disease included hypertension, depressive symptoms, traumatic brain injury, and family history of Parkinson's disease (n=310,866). The models for motor neurone disease included family history of dementia (n=325,870).

* Significant at a false discovery rate corrected significance level of 0.034

Supplementary Table 3. Subgroup analysis of the association between biological age measures and neurological disorders

Subgroup	KDMAge residual		PhenoAge residual		HDAge	
	HR per SD increase (95% CI)	$P_{interaction}$	HR per SD increase (95% CI)	$P_{interaction}$	HR per SD increase (95% CI)	$P_{interaction}$
**All-cause dementia**						
Age <60 (n=154,828)	1.32 (1.14, 1.54)	0.062	1.41 (1.23, 1.62)	0.005	1.13 (0.96, 1.32)	0.389
Age ≥60 (n=114,462)	1.16 (1.08, 1.24)		1.17 (1.09, 1.25)		1.07 (0.99, 1.16)	
Women (n=143,956)	1.17 (1.07, 1.29)	0.771	1.32 (1.21, 1.45)	0.029	1.21 (1.08, 1.35)	0.037
Men (n=125,334)	1.19 (1.10, 1.30)		1.13 (1.05, 1.22)		1.00 (0.91, 1.10)	
**Alzheimer's disease**						
Age <60 (n=154,828)	1.21 (0.92, 1.60)	0.489	1.42 (1.12, 1.80)	0.059	0.87 (0.64, 1.18)	0.443
Age ≥60 (n=114,462)	1.05 (0.94, 1.18)		1.08 (0.97, 1.20)		0.95 (0.84, 1.08)	
Women (n=143,956)	1.12 (0.96, 1.30)	0.442	1.30 (1.13, 1.50)	0.009	1.03 (0.86, 1.22)	0.128
Men (n=125,334)	1.03 (0.89, 1.19)		0.98 (0.85, 1.12)		0.87 (0.74, 1.02)	
**Vascular dementia**						
Age <60 (n=154,828)	1.96 (1.39, 2.76)	0.010	2.03 (1.47, 2.80)	0.002	1.50 (0.98, 2.31)	0.232
Age ≥60 (n=114,462)	1.35 (1.18, 1.54)		1.28 (1.13, 1.46)		1.22 (1.05, 1.42)	
Women (n=143,956)	1.28 (1.02, 1.61)	0.666	1.33 (1.08, 1.65)	0.611	1.29 (0.99, 1.67)	0.414
Men (n=125,334)	1.50 (1.30, 1.74)		1.35 (1.17, 1.56)		1.22 (1.03, 1.45)	
**Ischemic stroke**						
Age <60 (n=160,957)	1.37 (1.27, 1.49)	0.511	1.39 (1.29, 1.50)	0.125	1.28 (1.18, 1.39)	0.493
Age ≥60 (n=119,476)	1.40 (1.33, 1.47)		1.37 (1.31, 1.44)		1.26 (1.19, 1.34)	
Women (n=151,275)	1.30 (1.21, 1.40)	0.127	1.35 (1.26, 1.45)	0.911	1.35 (1.25, 1.47)	0.012
Men (n=129,158)	1.46 (1.38, 1.54)		1.40 (1.33, 1.47)		1.23 (1.16, 1.30)	
**Parkinson's disease**						
Age <60 (n=178,385)	0.87 (0.71, 1.07)	0.368	0.86 (0.70, 1.05)	0.343	0.78 (0.63, 0.97)	0.241
Age ≥60 (n=132,481)	0.97 (0.89, 1.07)		0.97 (0.89, 1.07)		0.90 (0.81, 1.00)	
Women (n=167,679)	0.99 (0.87, 1.13)	0.288	1.02 (0.90, 1.17)	0.090	0.99 (0.85, 1.15)	0.025
Men (n=143,187)	0.94 (0.84, 1.04)		0.91 (0.82, 1.01)		0.81 (0.72, 0.92)	
**Motor neurone disease**						
Age <60 (n=187,275)	1.26 (1.00, 1.59)	0.133	1.18 (0.94, 1.48)	0.048	1.26 (1.00, 1.57)	0.577
Age ≥60 (n=138,595)	1.06 (0.88, 1.28)		0.96 (0.80, 1.16)		1.19 (0.98, 1.44)	
Women (n=176,631)	1.12 (0.89, 1.40)	0.801	1.03 (0.82, 1.30)	0.847	1.21 (0.96, 1.54)	0.969
Men (n=149,239)	1.15 (0.95, 1.39)		1.05 (0.87, 1.26)		1.23 (1.02, 1.48)	

CI, confidence interval; *HDAge*, homeostatic dysregulation age; *HR*, hazard ratio; *KDMAge*, Klemera-Doubal method age; *SD*, standard deviation. All models were disease-specific models adjusted for the covariates listed in **Supplementary Table 1**. $P_{interaction}$ represent p-values of the multiplicative interaction terms between the continuous biological age measures and the subgroup indicator.

BMJ Publishing Group Limited (BMJ) disclaims all liability and responsibility arising from any reliance placed on this supplemental material which has been supplied by the author(s)

Supplementary Table 4. Association between biological age measures and neurological disorders after excluding diagnoses within 5 years from baseline

Model	KDMAge residual		PhenoAge residual		HDAge	
	HR per SD increase (95% CI)	*p*	HR per SD increase (95% CI)	*p*	HR per SD increase (95% CI)	*p*
**All-cause dementia**						
Age- & sex-adjusted model[a]	1.25 (1.18, 1.33)*	1.5×10^{-13}	1.26 (1.19, 1.33)*	1.6×10^{-14}	1.17 (1.10, 1.26)*	2.8×10^{-6}
Multivariable model[b]	1.25 (1.17, 1.33)*	6.2×10^{-12}	1.22 (1.15, 1.30)*	1.8×10^{-10}	1.17 (1.09, 1.25)*	1.4×10^{-5}
Disease-specific model[c]	1.17 (1.09, 1.26)*	2.9×10^{-5}	1.17 (1.09, 1.26)*	1.9×10^{-5}	1.05 (0.97, 1.14)	0.26
**Alzheimer's disease**						
Age- & sex-adjusted model[a]	1.11 (1.01, 1.23)	0.033	1.17 (1.06, 1.29)*	0.001	1.06 (0.95, 1.18)	0.326
Multivariable model[b]	1.12 (1.01, 1.24)	0.034	1.15 (1.04, 1.27)*	0.008	1.06 (0.95, 1.19)	0.292
Disease-specific model[c]	1.08 (0.96, 1.22)	0.209	1.13 (1.00, 1.27)	0.042	0.96 (0.84, 1.10)	0.591
**Vascular dementia**						
Age- & sex-adjusted model[a]	1.66 (1.48, 1.86)*	1.5×10^{-17}	1.54 (1.38, 1.73)*	1.5×10^{-13}	1.46 (1.27, 1.68)*	1.3×10^{-7}
Multivariable model[b]	1.61 (1.42, 1.82)*	6.1×10^{-14}	1.46 (1.29, 1.65)*	1.2×10^{-9}	1.40 (1.21, 1.62)*	6.0×10^{-6}
Disease-specific model[c]	1.42 (1.22, 1.64)*	3.8×10^{-6}	1.29 (1.12, 1.50)*	5.5×10^{-4}	1.20 (1.00, 1.43)	0.044
**Ischemic stroke**						
Age- & sex-adjusted model[a]	1.49 (1.42, 1.56)*	1.8×10^{-56}	1.46 (1.40, 1.54)*	3.6×10^{-57}	1.31 (1.24, 1.38)*	9.5×10^{-21}
Multivariable model[b]	1.44 (1.37, 1.52)*	4.6×10^{-41}	1.41 (1.34, 1.48)*	5.0×10^{-40}	1.25 (1.17, 1.32)*	3.0×10^{-13}
Disease-specific model[c]	1.37 (1.28, 1.45)*	1.2×10^{-22}	1.35 (1.28, 1.43)*	5.5×10^{-24}	1.19 (1.11, 1.27)*	1.2×10^{-6}
**Parkinson's disease**						
Age- & sex-adjusted model[a]	1.01 (0.92, 1.11)	0.842	0.98 (0.89, 1.07)	0.643	0.92 (0.83, 1.02)	0.133
Multivariable model[b]	1.01 (0.92, 1.12)	0.819	0.99 (0.90, 1.09)	0.800	0.91 (0.81, 1.01)	0.075
Disease-specific model[c]	1.01 (0.91, 1.11)	0.920	0.98 (0.89, 1.09)	0.738	0.89 (0.79, 1.00)	0.047
**Motor neurone disease**						
Age- & sex-adjusted model[a]	1.12 (0.93, 1.35)	0.223	1.10 (0.92, 1.32)	0.301	1.11 (0.92, 1.36)	0.281
Multivariable model[b]	1.11 (0.91, 1.35)	0.306	1.07 (0.88, 1.30)	0.477	1.11 (0.90, 1.36)	0.341
Disease-specific model[c]	1.11 (0.91, 1.35)	0.304	1.07 (0.88, 1.30)	0.475	1.11 (0.90, 1.36)	0.339

CI, confidence interval; *HDAge*, homeostatic dysregulation age; *HR*, hazard ratio; *KDMAge*, Klemera-Doubal method age; *SD*, standard deviation.

[d] Age- & sex-adjusted models: adjusted for age (timescale), birthyear, and sex (n=325,459).

[e] Multivariable models: additionally adjusted for baseline assessment centre, ethnicity, body mass index, smoking, alcohol consumption, and deprivation (n=325,459).

[f] Disease-specific models further included covariates that are relevant for each outcome based on the literature (**Supplementary Table 1**). The models for all-cause dementia, Alzheimer's disease, and vascular dementia included education, physical activity, social isolation, air pollution, diabetes, hypertension, depressive symptoms, hearing impairment, traumatic brain injury, *ApoE* e4 allele, and family history of dementia (n=269,000). The models for ischaemic stroke included physical activity, air pollution, fresh vegetable and fruit intake, red meat intake, processed meat intake, diabetes, hypertension, depressive symptoms, dyslipidaemia, atrial fibrillation, and family history of stroke (n=279,344). The models for Parkinson's disease included hypertension, depressive symptoms, traumatic brain injury, and family history of Parkinson's disease (n=325,644). The models for motor neurone disease included family history of dementia (n=325,777).

* Significant at a false discovery rate corrected significance level of 0.025
